# Exogenous Physical Irradiation on Titania Semiconductors: Materials Chemistry and Tumor‐Specific Nanomedicine

**DOI:** 10.1002/advs.201801175

**Published:** 2018-10-17

**Authors:** Ruifang Zhang, Fei Yan, Yu Chen

**Affiliations:** ^1^ Department of Ultrasound The First Affiliated Hospital of Zhengzhou University Zhengzhou Henan Province 450052 P. R. China; ^2^ Paul C. Lauterbur Research Center for Biomedical Imaging Institute of Biomedical and Health Engineering Shenzhen Institutes of Advanced Technology Chinese Academy of Sciences Shenzhen 518055 P. R. China; ^3^ State Key Laboratory of High Performance Ceramics and Superfine Microstructure Shanghai Institute of Ceramics Chinese Academy of Sciences Shanghai 200050 P. R. China

**Keywords:** cancer, nanomedicine, physical irradiation, semiconductors, titania

## Abstract

Titania semiconductors can be activated by external physical triggers to produce electrons (e^−^) and holes (h^+^) pairs from the energy‐band structure and subsequently induce the generation of reactive oxygen species for killing cancer cells, but the traditional ultraviolet light with potential phototoxicity and low‐tissue‐penetrating depth as the irradiation source significantly hinders the further in vivo broad biomedical applications. Here, the very‐recent development of novel exogenous physical irradiation of titania semiconductors for tumor‐specific therapies based on their unique physiochemical properties, including near infrared (NIR)‐triggered photothermal hyperthermia and photodynamic therapy, X‐ray/Cerenkov radiation‐activated deep‐seated photodynamic therapy, ultrasound‐triggered sonodynamic therapy, and the intriguing synergistic therapeutic paradigms by combined exogenous physical irradiations are in focus. Most of these promising therapeutic modalities are based on the semiconductor nature of titania nanoplatforms, together with their defect modulation for photothermal hyperthermia. The biocompatibility and biosafety of these titania semiconductors are also highlighted for guaranteeing their further clinical translation. Challenges and future developments of titania‐based therapeutic nanoplatforms and the corresponding developed therapeutic modalities for potential clinical translation of tumor‐specific therapy are also discussed and outlooked.

## 1 Introduction

As one of the mostly explored multidisciplinary research frontiers, nanomedicine has attracted the broad attention of scientific community ranging from material science, chemistry, pharmacy, biology, and biomedicine.^1^, ^2^, ^3^, ^4^, ^5^, ^6^ It has shown the intriguing performance and application prospect in molecular imaging for disease diagnosis, targeted drug delivery for enhanced chemotherapy, some physically triggered novel therapeutic modalities, diagnostic biosensing, and even tissue engineering.^7^, ^8^, ^9^, ^10^ Various nanoparticles with their intrinsic desirable composition, nanostructure, physiochemical property, and biological effects have been explored to achieve the efficient therapeutic performance and outcome in the past decades, among which inorganic nanoplatforms have been attracting the high research interest very recently because of their unique physiochemical property, multifunctionality (e.g., optical property, magnetism, electronic behavior and acoustic property) and relatively high biocompatibility.^11^, ^12^, ^13^, ^14^, ^15^, ^16^, ^17^

Organic nanosystems have been broadly investigated and some of them have entered the clinical stage for benefiting the patients.^1^, ^18^, ^19^, ^20^, ^21^ It is noted that the organic nanosystems typically lack the functionality, which means that they cannot be easily designed for some unique and specific theranostic purposes. Comparatively, inorganic nanosystems can be facilely endowed with specific properties of magnetism, fluorescence, ultrasound responsiveness, electronic conductivity, etc. They can also be designed with some intriguing nanostructures and topologies. For instance, the mostly explored mesoporous silica nanoparticles (MSNs) are fabricated with well‐defined mesoporous nanostructure, which provide the large reservoirs for the efficient loading and delivery and therapeutic guest molecules.^22^, ^23^ Another paradigm of inorganic nanoparticles is the mostly studied superparamagnetic iron oxide nanoparticles (SPIONs) for contrast‐enhanced magnetic resonance imaging (MRI), magnetically targeted drug delivery and magnetic hyperthermia, which are all based on their intriguing magnetic properties.^24^, ^25^, ^26^ Especially, the plasmonic resonance property of gold (Au) nanoparticles has been adopted for photo‐triggered hyperthermia, computed tomography (CT) imaging, and biosensing applications.^27^, ^28^

Compared to mostly explored metal oxides such as silica, manganese oxide, and iron oxide nanoparticles, titania nanosystems have emerged as a novel inorganic nanoplatform with their intrinsic physiochemical properties suitable for biomedical applications.^29^, ^30^, ^31^, ^32^, ^33^, ^34^, ^35^ Titanium (Ti) has been demonstrated as one of the biocompatible elements. For instance, titanium oxide (TiO_2_) has been extensively used in colorant in food,^36^, ^37^, ^38^ cosmetics,^39^, ^40^, ^41^ and sunscreen.^42^, ^43^, ^44^, ^45^, ^46^ Especially, Ti‐containing metal alloys have been employed as the medical implantation devices.^47^, ^48^, ^49^, ^50^, ^51^, ^52^, ^53^ As one of the mostly explored semiconductors, traditional TiO_2_ nanoparticles have a bandgap of 3.2 eV, which can be excited by ultraviolet (UV) light.^54^, ^55^, ^56^, ^57^ The UV light with radiative energy higher than its bandgap of 3.2 eV (387 nm) excites an electron to the conduction band (CB) and creates an electron hole pair.^58^, ^59^, ^60^, ^61^ The produced electrons can reduce the absorbed molecular oxygen to superoxide radicals, and the holes are capable of oxidizing the water molecules into hydrogel radicals (•OH). Therefore, TiO_2_ nanoparticles can function as the inorganic photosensitizers to produce large amounts of reactive oxygen species (ROS) for photodynamic therapy (PDT).^62^, ^63^, ^64^, ^65^, ^66^, ^67^ Especially, the semiconductor nature and unique photoresponsiveness of TiO_2_ have been employed for the degradation of organic substrates^68^, ^69^, ^70^ and deactivation of microorganisms/viruses.^71^, ^72^, ^73^, ^74^

**Figure 1:**
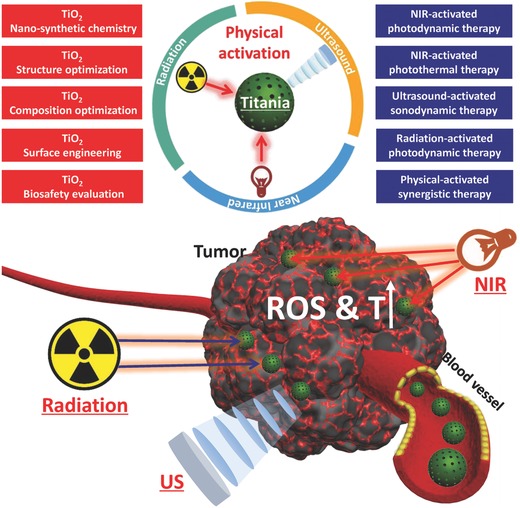
Schematic illustration of exogenous physical activation of titania nanoparticles for tumor‐specific therapy. It includes NIR‐activated PDT/PTT, radiation‐activated PDT, US‐activated SDT and physical activation‐based synergistic therapy. The related research frontiers are also summarized in the figure, such as nano‐synthetic chemistry for titania fabrication, structure/composition optimization, surface engineering, and biosafety evaluation.

However, traditional titania nanoparticles only respond to UV light, which unfortunately has potential phototoxicity and low tissue‐penetrating depth, severely hindering their further clinical translation.^29^, ^75^ Very recently, the fast development of theranostic nanomedicine has explored more effective exogenous physical triggers for activating titania nanoparticles for achieving some specific but intriguing therapeutic modalities, such as near infrared (NIR)‐triggered photothermal therapy (PTT), NIR‐activated PDT, X‐ray/Cerenkov radiation (CR)‐activated deep‐seated PDT, and US‐triggered SDT (**Figure** **1**). All these exogenous physical triggers are featured with their intrinsic characteristics but also with some drawbacks, which is the main topic of this review. Therefore, this review discusses the very‐recent progresses of adopting novel exogenous physical irradiations to activate titania semiconductors for some specific tumor therapies, which are mainly based on the semiconductor nature of titania nanosystems accompanied with the unique oxygen‐defect modulation for photothermal hyperthermia. In addition, the critical issue of biocompatibility and biosafety of these titania nanoplatforms has also been discussed to potentially guarantee the further clinical translation. Finally, the critical challenges and further future developments of the novel physical‐triggered titania‐based nanoplatforms and the corresponding intriguing therapeutic modalities are also deeply discussed to promote the progress of this novel inorganic nanoplatform in theranostic nanomedicine.

## 2 Synthesis, Multifunctionalization, and Surface Engineering of Titania Nanoparticles in Biomedicine

The rational design and successful construction of titania‐based nanoplatforms are the bases for achieving the high theranostic performance in biomedicine, which is mostly based on the advances of nanosynthetic chemistry and material chemistry.^76^, ^77^, ^78^, ^79^, ^80^, ^81^, ^82^, ^83^ We recently synthesized highly dispersed mesoporous titania nanoparticles (MTNs) with monodispersity and uniformity by a method of prehydrolysis of titanium precursors combined with solvothermal treatment, which provided both the high crystallized framework and well‐defined mesoporous nanostructure.^84^ In addition, we recently synthesized oxygen‐deficient core/shell‐structured black TiO_2−_ *_x_* nanoparticles by a facile aluminum reduction methodology for enhanced sonodynamic therapy (SDT) and simultaneous NIR‐triggered photothermal hyperthermia at NIR‐II biowindow.^85^ Especially, mesoporous black TiO_2−_ *_x_* nanoparticles were fabricated by a two‐step procedure where the mesoporous white TiO_2_ nanoparticles were initially synthesized, followed by reduction under hydrogen atmosphere at 500 °C for 1 h to turn white TiO_2_ into black TiO_2−_ *_x_*.^86^

Especially, the advances of nanosynthetic chemistry make the precise controlling of titania's composition, nanostructure and functionality possible where these titania nanocomponents can also be integrated with other functional moieties or nanoparticles to achieve some specific purposes. For instance, the NaYF_4_:Yb,Tm nanoparticles were initially coated by a silica layer with the grafting of (3‐aminopropyl)‐trimethoxysilane, which provided the positively charged amino groups for efficient binding titanium precursors, guaranteeing the gradual epitaxial growth of uniform TiO_2_ layer onto the surface of initially synthesized nanoparticles.^87^ NaYF_4_:Yb^3+^,Tm^3+^@NaGdF_4_:Yb^3+^@TiO_2_ (UCNPs@TiO_2_) core/shell nanoparticles were synthesized by direct in situ growth protocol. NaYF_4_:Yb^3+^,Tm^3+^@NaGdF_4_:Yb^3+^ were initially synthesized, followed by surface modification with polyvinylpyrilidone (PVP).^88^ Then, TiF_4_ acted as the Ti precursors for the direct formation of TiO_2_ nanoshells on the surface of UCNPs by the hydrolysis and condensation process. This one‐step PVP‐mediated methodology is facile and generic for the construction of TiO_2_‐based nanocomposites, especially for the construction of UCNPs@TiO_2_ composite nanoplatforms.^89^

For TiO_2_‐based functionalization, we also directly grew TiO_2_ nanoparticles onto the surface of graphene oxide (GO) by a hydrothermal treatment of the cosolvent solution of TiO_2_ nanoparticles and GO suspension, based on which TiO_2_ nanoparticles were uniformed dispersed onto the surface of 2D planar GO.^90^ Au–TiO_2_ nanocomposites were synthesized by a facile photoreduction of Au^3+^ ions, which could be deposited on the surface of TiO_2_ nanoparticles to grow Au nanoparticles under UV irradiation.^91^ The particle size of deposited Au nanoparticles could be controlled by adopting the UV‐irradiation durations. Especially, the dumbbell‐like Au–TiO_2_ nanoparticles were synthesized by a seed‐mediated growth approach.^92^ Au nanoparticles were initially synthesized, acting as the growing sites for the TiO_2_ generation in an anisotropic manner by controlling the hydrolysis degree of introduced Ti precursor.

The surface chemistry of titania‐based nanoplatforms is also of high significance in biomedicine. For instance, the adequate surface modification can either improve the stability of these nanoparticles in physiological solution or achieve the positive‐targeting accumulation into tumor cells/tissues. The surface of Au–TiO_2_ nanocomposites was modified with biocompatible carboxymethyl dextran (CMD) for achieving prolonged systemic circulation and subsequent enhanced tumor‐homing capability.^91^ We modified the surface of black TiO_2−_ *_x_* nanoparticles with NH_2_–PEG_2000_ molecules by a simple sonication procedure, which was based on the coordination interaction between N component of PEG molecules and Ti atoms of black TiO_2_ nanoparticles.^85^ We also modified the surface of TiO_2_‐loaded GO by PVP molecules for enhanced stability in physiological condition, which could guarantee the further in vivo biomedical applications on combating cancer.^90^ In addition, the precoating of SiO_2_ layer onto the surface of TiO_2_‐based nanocomposite could provide the anchoring sites of silane group of maleimide‐PEG‐silane, achieving the efficient PEGylation of TiO_2_‐based nanocomposites.^87^ Especially, anti‐cAngptl4 Ab was conjugated onto the surface of N‐TiO_2_/NaYF_4_:Yb,Tm nanocomposites for targeted cancer‐cell PDT on killing cancer cells as induced by NIR irradiation.^93^

## 3 Light Irradiation on Titania for PDT

PDT on combating cancer is featured with noninvasiveness and tumor specificity, which typically employs the external physical light source for activating photosensitizers to produce toxic ROS and consequently kill the cancer cells.^94^, ^95^, ^96^, ^97^ Light‐excited PDT has been clinically used for the treatment of cancers on skin and other epidermal tissues. As compared to traditional organic photosensitizers, TiO_2_‐based inorganic nano‐photosensitizers are featured with high stability and nontoxicity, which has shown broad application potentials in PDT‐based cancer treatment.^98^, ^99^, ^100^, ^101^, ^102^, ^103^, ^104^, ^105^, ^106^, ^107^, ^108^

**Figure 2:**
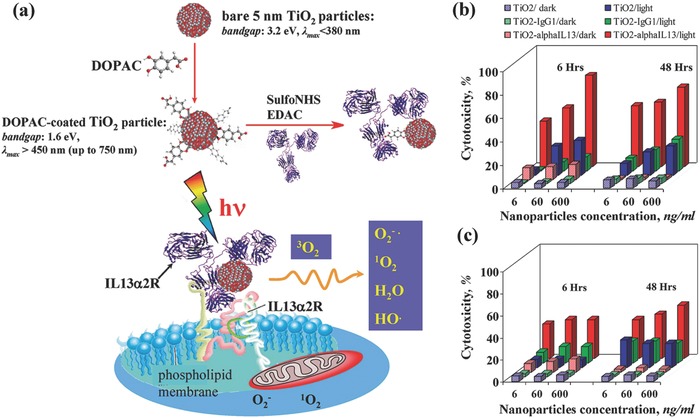
a) Schematic illustration of the fabrication of titania nanoparticles with surface‐linked IL13R‐recognizing antibody, and their further recognition and binding to surface IL13R of cancer cell for visible light‐activated ROS generation and subsequently inducing cancer‐cell death. In vitro therapeutic efficiency by evaluating the phototoxicity of TiO_2_‐mAb against b) A172 GBM cells (high ILα2R expression) and c) U87 GBM cells (low ILα2R expression). Reproduced with permission.^109^ Copyright 2009, American Chemical Society.

Based on titania nanoparticles, a polychromatic visible light‐activated nano‐biohydrid system was constructed by covalently binding an antibody via a dihydroxybenzene bivalent linker that could selectively recognize glioblastoma multiforme (GBM) cells (**Figure** **2**a).^109^ This targeting strategy enhanced the intracellular uptake of TiO_2_ nanoparticles and produced large amounts for ROS for damaging the cell membrane, inducing the cancer‐cell death under the visible‐light irradiation (Figure 2b,c). The PDT efficiency of titania could also be achieved by heterogeneous atom doping. For instance, the Fe‐doping of TiO_2_ nanotubes was demonstrated to realize near‐visible light‐driven (2.30 mW cm^−2^, ≈405 nm) PDT on killing cervical cancer cells, and the phototoxicity of Fe‐doped TiO_2_ nanotubes was much higher than that of undoped TiO_2_ nanotubes.^103^ Furthermore, nitrogen‐doped TiO_2_ (N‐TiO_2_) also showed visible light‐triggered PDT against HeLa cancer cells where the N‐doped TiO_2_ nanoparticles were featured with higher PDT efficiency as compared to that of pure TiO_2_ nanoparticles.^110^ The related mechanism investigation revealed that N‐TiO_2_ induced more loss of mitochondrial membrane potential and higher increase of intracellular Ca^2+^ and nitrogen monoxide in HeLa cancer cells than pure TiO_2_ nanoparticles.

**Figure 3:**
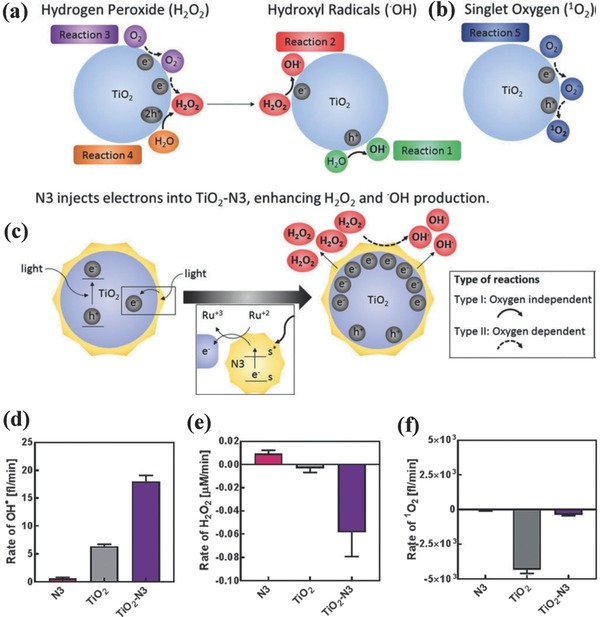
Schematic illustration of ROS generation, including a) H_2_O_2_ production and transformation into hydroxyl radical, b) singlet oxygen (^1^O_2_) production, and c) electron injection by N3 into TiO_2_–N3 to enhance H_2_O_2_ and hydroxyl radical production. The generation rate of d) hydroxyl radical, e) H_2_O_2,_ and f) singlet oxygen under hypoxic conditions. Reproduced with permission.^111^ Copyright 2017, WILEY‐VCH Verlag GmbH & Co. KGaA, Weinheim.

To further enhance the PDT efficiency of TiO_2_‐based photosensitizers, TiO_2_ nanoparticles were conjugated with ruthenium complex (N3) for improved and synergistic production of ROS in both hypoxic and normoxic conditions.^111^ By light irradiation (365 nm), the N3 injected electrons into TiO_2_ nanoparticles, resulting in the production of three‐ and fourfold more hydroxyl radicals (•OH) and hydrogen peroxide (H_2_O_2_) as compared to bare TiO_2_ nanoparticles, respectively. Bare TiO_2_ nanoparticles could oxidize water molecules to produce hydroxyl radical (•OH, **Figure** **3**a). The presence of light‐induced electron–hole pair in TiO_2_ facilitated the reduction of molecular oxygen to superoxide and then transformation to single oxygen (^1^O_2_, Figure 3b). Especially, under the hypoxic condition, the N3 facilitated the electron–hole reduction of absorbed water molecules to enhance the hydroxyl radical production with nearly threefold increase (Figure 3c,d). This strategy could transform TiO_2_ photosensitizer from a dual type I and II PDT nanoagents into a mainly type I photosensitizer independent of the oxygen level (Figure 3d–f).^111^ This work provides an efficient strategy to enhance the TiO_2_‐based PDT efficiency in hypoxic condition by N3 hybridization. Coating a homogenous TiO_2_ layer onto the surface of ZnTPyP self‐assembly nanocrystal achieved the photoelectron transfer at ZnTPyP self‐assembly/TiO_2_ interfaces, which further enhanced the two‐photon PDT against HeLa cancer cells via type‐1‐like PDT process.^65^ This titania‐based composite nanoplatform is very intriguing because the achieved two‐photon PDT is highly desirable for deep‐tissue disease treatment.^96^, ^112^

**Figure 4:**
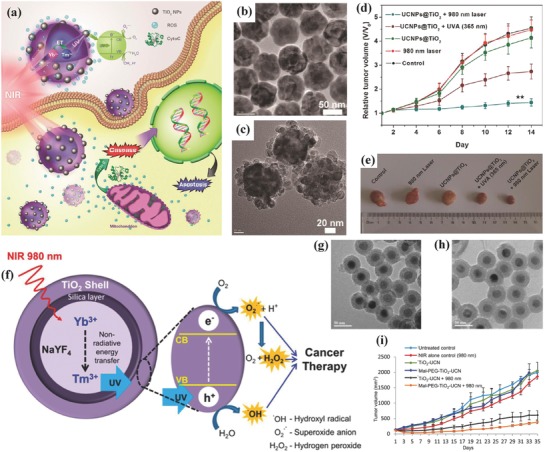
a) Schematic illustration of NIR‐triggered PDT based on UCNPs@TiO_2_‐based nano‐photosensitizers and the underlying therapeutic mechanism including the induced apoptosis of cancer cells. TEM images of b) NaYF_4_:Yb^3+^,Tm^3+^@NaGdF_4_:Yb^3+^ core/shell nanoparticles and c) NaYF_4_:Yb^3+^,Tm^3+^@NaGdF_4_:Yb^3+^@TiO_2_ (UCNPs@TiO_2_) core/shell nanoparticles. d) The tumor‐volume changes of tumor‐bearing mice in varied treatment groups as shown in the figure, and e) the corresponding digital photographic images of excised tumors at the end of treatments. Reproduced with permission.^88^ Copyright 2015, American Chemical Society. f) The scheme of UCNP‐mediated activation of surface‐coated TiO_2_ layer for ROS production to kill the cancer cells. TEM images of g) TiO_2_‐UCNPs and h) Mal‐PEG‐TiO_2_‐UCNPs. i) The in vivo OSCC tumor‐growth volumes within the 35 d duration after varied treatments as indicated in the figure. Reproduced with permission.^87^ Copyright 2015, American Chemical Society.

The major challenge of TiO_2_‐based PDT is the light responsiveness only in the wavelength range of UV or visible light, which has the low tissue‐penetrating distance and causes the failure in the treatment of deep‐seated tumor. Upconversion nanoparticles (UCNPs) are capable of generating high energy light from the low energy light such as NIR light.^113^, ^114^, ^115^ Therefore, lanthanide‐doped UCNPs can convert NIR light into UV or visible photons via an anti‐Stokes emission process, which potentially acts as the “nano‐transducers” to achieve NIR‐triggered PDT.^116^, ^117^, ^118^ On this ground, core/shell‐structured UNCPs (NaYF_4_:Yb^3+^,Tm^3+^@NaGdF_4_:Yb^3+^) with enhanced upconverting UV emission were initially synthesized, followed by coating with TiO_2_ shells using TiF_4_ as the Ti precursor to in situ grow TiO_2_ shells onto the surface of UNCPs under mild hydrolysis condition (**Figure** **4**a–c).^88^ The UCNPs core emitted upconverting light in UV/visible range by 980 nm NIR irradiation, which was substantially diminished by the absorbance of TiO_2_ shells in such a wavelength range (Figure 4a). Such an energy‐transferring process induced the extracellular and intracellular generation of ROS for causing the cancer‐cell death by inducing the cell apoptosis. HeLa tumor‐bearing model results showed that the intratumoral injection of NaYF_4_:Yb^3+^,Tm^3+^@NaGdF_4_:Yb^3+^@TiO_2_ (UCNPs@TiO_2_) core/shell nanoparticles followed by 980 nm laser irradiation achieved the substantial tumor‐growth suppression with high therapeutic efficiency/outcome (Figure 4d,e), which was further demonstrated by immunohistochemical staining for caspase 3.^88^

Similarly, Zhang and co‐workers coated a TiO_2_ layer onto the surface of SiO_2_‐coated UCNPs (NaYF_4_:20%Yb,0.5%Tm) for 980 nm NIR‐triggered PDT. The UCNPs core converted NIR irradiation into UV light, which photoexcited electrons in the valence band (VB) of TiO_2_ shell to the CB, forming the photo‐induced hole–electron pairs (Figure 4f).^87^ The postgenerated hole–electron pairs reacted with surrounding molecular oxygen and water molecules to generate ROS and then induce the cancer‐cell death. The TiO_2_‐coated UCNPs were clearly characterized by TEM image (Figure 4g,h). After the intratumoral injection of these composite nanoparticles followed by 980 nm NIR irradiation, the significant tumor‐growth suppression was achieved (Figure 4i). In fact, the TiO_2_‐coated UCNPs themselves are highly biocompatible, and only the NIR‐irradiated tumor region can produce toxic ROS, therefore their impact to normal cells and tissues are low, leading to high therapeutic biosafety. Furthermore, anti‐EGRF‐affibody was conjugated to PEGylated TiO_2_–UCNPs nanocomposites for targeting epithelial growth factor receptor (EGFR) overexpressing oral cancer cells and the subsequent NIR‐excited PDT with the therapeutic outcome of significantly suppressed tumor growth and improved survival rate of tumor‐bearing mice.^104^

**Figure 5:**
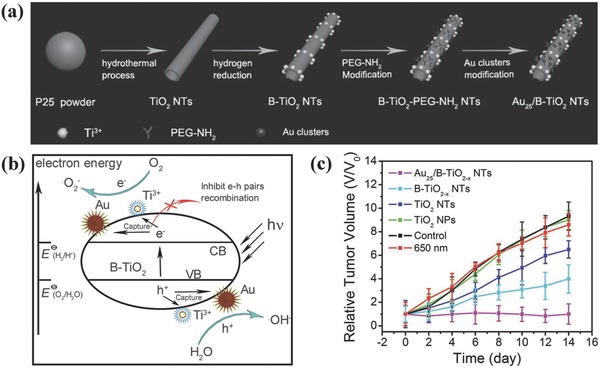
a) Schematic illustration of the synthetic process for Au_25_/B–TiO_2−_ *_x_* nanotubes. b) The scheme of photocatalytic mechanism for ROS production as assisted by the introduced Au_25_/B–TiO_2−_ *_x_* nanotubes. c) In vivo therapeutic outcome as indicated by the relative tumor‐volume changes after varied treatments. Reproduced with permission.^119^ Copyright 2017, WILEY‐VCH Verlag GmbH & Co. KGaA, Weinheim.

The photoresponsive wavelength range could also be controlled by rational design of the composition and nanostructure of titania‐based nanoplatforms. For instance, 808 nm NIR‐activation of black TiO_2_ nanoparticles with a narrow bandgap of around 2.32 eV was demonstrated to absorb NIR light and subsequently produce abundant ROS for photodynamic killing of bladder cancer cells.^120^ In addition, Au cluster‐anchored black anatase TiO_2−_ *_x_* nanotubes (designated as Au_25_/B‐TiO_2−_ *_x_*) were stepwise synthesized by gaseous hydrogen reduction of TiO_2_ nanotubes followed by the deposition of Au clusters (**Figure** **5**a).^119^ These Au_25_/B‐TiO_2−_ *_x_* exhibited the photo‐responsiveness in NIR range (650 nm) for PDT against cancer. The surface modification of Au clusters changed the electrical distribution in the composite nanosystem, which could reduce the recombination of electrons and holes as triggered by NIR irradiation (Figure 5b). Importantly, the hydrogen reduction generated large amount of Ti^3+^ ions in the matrix of black TiO_2‐x_, which extended the light response of anatase TiO_2_ nanoparticles from UV light to NIR light. In vivo therapeutic evaluation on tumor‐bearing xenograft revealed that the significantly enhanced therapeutic efficacy was achieved based on the photocatalytic synergistic effect, which substantially suppressed the tumor growth after the injection of Au_25_/B‐TiO_2−_ *_x_* followed by NIR irradiation (Figure 5c).

**Figure 6:**
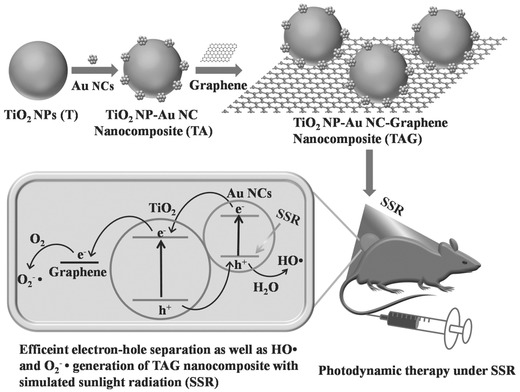
Schematic illustration of the fabrication of TAG composite nanoplatform and the related PDT mechanism for cancer therapy. Reproduced with permission.^121^ Copyright 2017, WILEY‐VCH Verlag GmbH & Co. KGaA, Weinheim.

To achieve simulated sunlight‐irradiated PDT, TiO_2_–Au–graphene (designated as TAG) heterogeneous nanocomposites were designed and fabricated for employing simulated sunlight as physical triggering source to kill melanoma skin cancer cells by photodynamic effect.^121^ The narrow bandgap of Au nanoclusters and staggered energy bands of Au–TiO_2_–graphene resulted in the efficient use of simulated sunlight, which also enhanced the separation efficiency of electron–hole pairs for producing large amounts of hydroxyl and superoxide radicals.^122^, ^123^, ^124^, ^125^, ^126^, ^127^ Typically, the sunlight‐excited electrons from HOMO to LUMO of Au nanoclusters were transferred to the conductive band of titania nanoparticles and then to the graphene matrix, which further acted as the free electrons and further generated superoxide radicals by reacting with oxygen molecules. The holes from both HOMO of Au nanoclusters and valance band of titania nanoparticles accumulated on HOMO of Au nanoclusters, which further reacted with water molecules to produce hydroxyl radicals (**Figure** **6**). These TGA nanocomposites have been demonstrated to trigger a series of toxicological effects on killing B16F1 melanoma cells against B16F1 tumor xenograft, indicating high photodynamic efficiency of this prominent therapeutic modality for sunlight‐triggered PDT effect. Although above‐mentioned paradigms are effective on phototriggered PDT for cancer therapy based on TiO_2_‐based photosensitizers, this therapeutic modality is still suffering from the low tissue‐penetrating capability of light as the irradiation source.

## 4 Laser Irradiation on Titania for PTT

In addition to NIR‐triggered PDT for combating cancer, NIR‐induced PTT has emerged as an efficient therapeutic modality for tumor treatment.^28^, ^128^, ^129^, ^130^, ^131^ Typically, the exogenous NIR laser can penetrate through the skin and activate the photothermal agents for converting NIR energy into heat and then ablating the tumor tissue by simply elevating the tumor temperature subsequently.^132^, ^133^, ^134^, ^135^, ^136^ Therefore, the development of desirable photothermal‐conversion agents plays the determining role for achieving the efficient and desirable PTT outcome.^137^, ^138^, ^139^, ^140^, ^141^, ^142^, ^143^, ^144^, ^145^, ^146^

**Figure 7:**
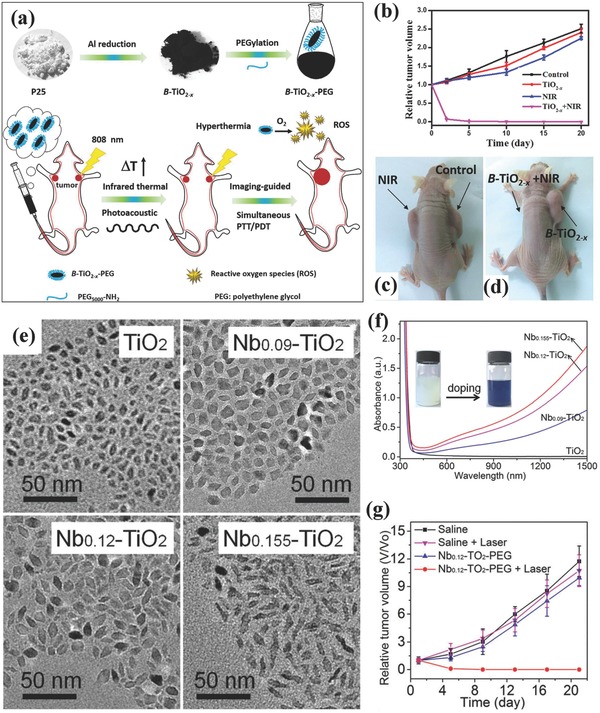
a) Schematic illustration of synthesizing PEGylated black TiO_2−_ *_x_* nanoparticles and their unique functionality for PA imaging‐guided photothermal hyperthermia of tumor under NIR laser irradiation. b) The relative tumor‐volume changes after varied treatments including control group, NIR group, TiO_2−_ *_x_* group and TiO_2−_ *_x_* combined with NIR irradiation group. c,d) Photographic image of tumor at the end of each treatment. Reproduced with permission.^147^ Copyright 2016, Elsevier. e) TEM images of TiO_2_ nanoparticles with varied Nb‐doping amount. f) UV–vis–NIR absorbance spectra of Nb‐doped TiO_2_ nanoparticles in chloroform. g) The relative HeLa tumor‐volume changes as a function of feeding time after different treatments as shown in the figure. Reproduced with permission.^148^ Copyright 2017, Royal Society of Chemistry.

Based on an ambient heterogeneous spark discharge, Au–TiO_2_ heterodimers were fabricated by incorporating Au component into TiO_2_ nanoparticles, which exhibited the visible light‐induced photothermal effect on killing HeLa cancer cells based on the localized surface plasmon resonance of integrated ultrafine Au nanoparticles.^149^ Al reduction could transform white P25‐type TiO_2_ nanoparticles into oxygen‐deficient black TiO_2−_ *_x_* (B‐TiO_2−_ *_x_*) nanoparticles,^150^, ^151^, ^152^ which endowed these black TiO_2−_ *_x_* nanoparticles with unique photothermal‐conversion capability for efficient photothermal hyperthermia of cancer. Mo et al. modified the surface of B‐TiO_2−_ *_x_* nanoparticles with PEG molecules for guarantee their high stability in physiological condition (**Figure** **7**a). After intravenous administration into HeLa tumor‐bearing mice, these PEGylated B‐TiO_2−_ *_x_* nanoparticles efficiently accumulated into tumor tissue and rapidly elevated the tumor temperature by 808 nm NIR irradiation, causing the complete photothermal eradication of tumor tissue (Figure 7b–d).^147^ Besides the Al reduction to fabricate black TiO_2−_ *_x_* nanoparticles, the hydrogenated black TiO_2_ (H‐TiO_2_) nanoparticles also exhibited high NIR absorption, which were further developed as the photothermal‐conversion nanoagents for efficient tumor photohyperthermia based on their high photothermal‐conversion efficiency of as high as 40.8% at the wavelength of 808 nm.^153^

In addition to the mostly explored high‐temperature treatment strategy to obtain black TiO_2_ nanoparticle for PTT against cancer, Chen and co‐workers successfully converted UV‐responsive TiO_2_ nanoparticles to blue TiO_2_ nanocrystals by a simple Nb‐doping approach.^148^ The different Nb‐doping amount induced varied morphology of TiO_2_ nanocrystals with high dispersity (Figure 7e). Especially, the efficient Nb‐doping endowed these blue TiO_2_ with the strong NIR absorbance (Figure 7f), which was originated from the localized surface plasmon resonances because of Nb doping‐induced considerable free electrons. These blue Nb‐doped TiO_2_ nanocrystals efficiently converted laser at NIR‐II biowindow (1064 nm) into heat and induced the photothermal effect on ablating the tumor tissue with high PTT efficiency (Figure 7g).^148^

**Figure 8:**
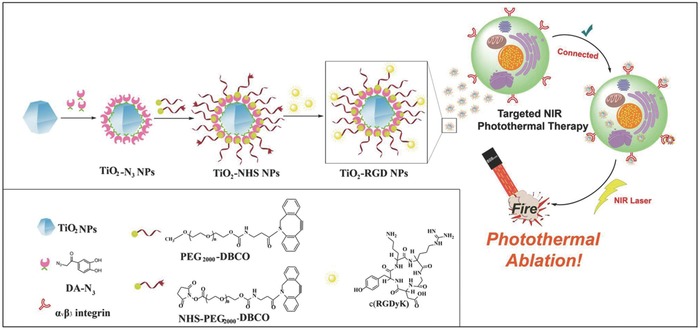
The scheme of the synthetic procedure of targeted TiO_2_–RGD nanoparticles and their unique functionality for NIR‐triggered photothermal hyperthermia against α_v_β_3_ integrin‐overexpressed cancer cells. Reproduced with permission.^154^ Copyright 2017, Springer Nature Publishing Group.

The endowed targeting property of titania nanoparticles potentially enhances the tumor‐accumulation efficiency for improved cancer therapy. On this ground, the surface of NIR‐responsive TiO_2_ nanoparticles as the photothermal‐conversion nanoagents was conjugated with cyclo(Arg‐Gly‐Asp‐d‐Tyr‐Lys) peptide c(RGDyK) for targeted photothermal hyperthermia of cancer (**Figure** **8**).^154^ Based on the absorption of electron localized on Ti(III) sites and free electrons existing in the conduction bond, these TiO_2_ nanoparticles showed high photothermal‐conversion efficiency of nearly 38.5%. The surface‐modified c(RGDyK) peptide selectively targeted the α_v_β_3_ integrin on the cancer‐cell membrane (U87‐MG human glioblastoma cells) for efficiently killing the cancer cells, demonstrating the effectiveness of targeting strategy for improving the therapeutic efficiency of PTT.^154^

## 5 Radiation‐Activated Titania for PDT

The traditional external laser‐activated PDT or PTT still suffers from the low tissue‐penetrating depth of laser because of the rapid light attenuation passing through tissue and difficulty for reaching the deep‐seated malignant lesions, which only confines the photointerventions for the treatment of superficial diseases.^155^ Radiation therapy by using radiation sources can solve above‐mentioned critical issue because of the high tissue‐penetrating capability of these radiation sources.^156^, ^157^, ^158^, ^159^, ^160^, ^161^ Especially, the advances of theranostic nanomedicine has demonstrated the augmenting effect of some nanoparticulate radiosensitizers for substantially enhanced radiation‐therapy outcome.^162^, ^163^, ^164^, ^165^, ^166^, ^167^, ^168^, ^169^, ^170^ Therefore, recent advances have also revealed the possibility of exogenous physical radiation sources for activating titania‐based nanoplatforms to achieve efficient cancer therapy.

**Figure 9:**
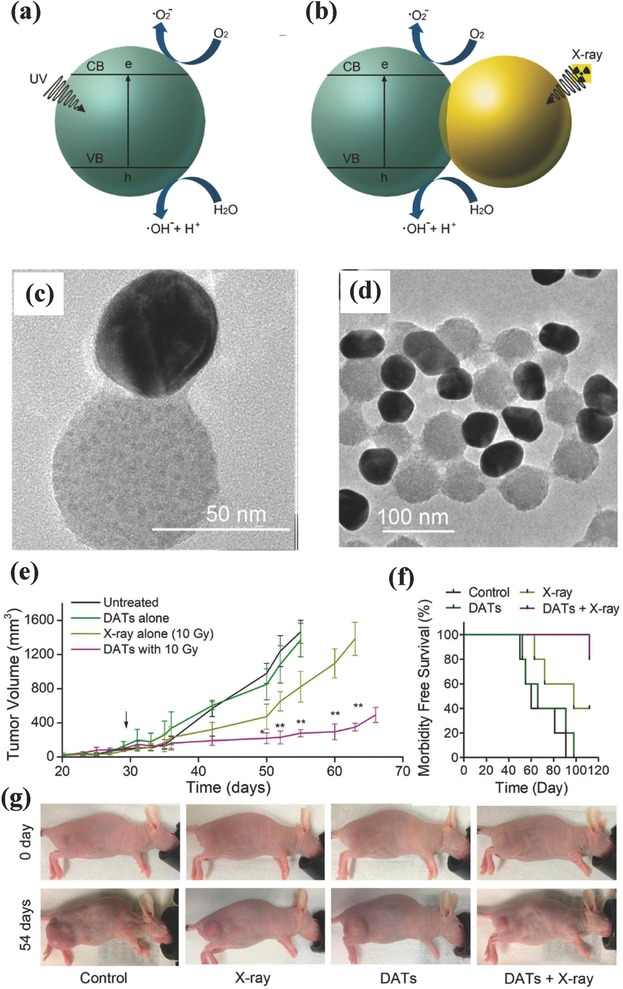
The scheme of ROS production on a) photoactivated TiO_2_ nanoparticles and b) X‐ray‐induced hybrid DATs. TEM images of hybrid DATs at different magnifications. c) In vivo tumor‐volume changes of tumor‐bearing xenograft after different treatments as indicated in the figure, and d) corresponding survival rate of SUM159‐tumor‐bearing mice after varied treatments. e) Photographic images of SUM159 tumor‐bearing mice before and at the end of treatments. f) The survival rate of tumor‐bearing mice after varied treatments, and g) corresponding representative photographic images of mice before and after different treatments. Reproduced with permission.^92^ Copyright 2018, American Chemical Society.

Au and titania anisotropic nanostructure was rationally designed as radio‐sensitizers for X‐ray‐activated radiation therapy.^92^ Typically, bare TiO_2_ nanoparticles could generate cytotoxic hydroxyl and superoxide radicals by the activation of UV light (**Figure** **9**a). Dumbbell‐like Au–TiO_2_ nanoparticles (DATs) can be activated by ionizing radiation and then produce secondary photons or electrons,^171^, ^172^, ^173^ which could induce the ROS production and migrate over the interface of DAT to TiO_2_ component for further ROS production on the surface of TiO_2_ (Figure 9b). The anisotropic nanostructure of DATs was constructed by stepwise seed‐mediated growth (Figure 9c,d). Based on the strong asymmetric electric coupling between Au component and dielectric TiO_2_ at the interface, these DATs exhibited a synergistic therapeutic efficiency on X‐ray‐triggered radiation therapy where the production of secondary electrons and ROS from DATs substantially enhanced the radiation effect, causing the high tumor‐suppressing effect (Figure 9e–g) and survival rate of tumor‐bearing mice (Figure 9f).^173^ This paradigm demonstrates that the rational integration of TiO_2_ nanoparticles with functional nanoparticles can significantly enhance the efficiency of radiation therapy by taking the unique characteristics of each integrated component.

**Figure 10:**
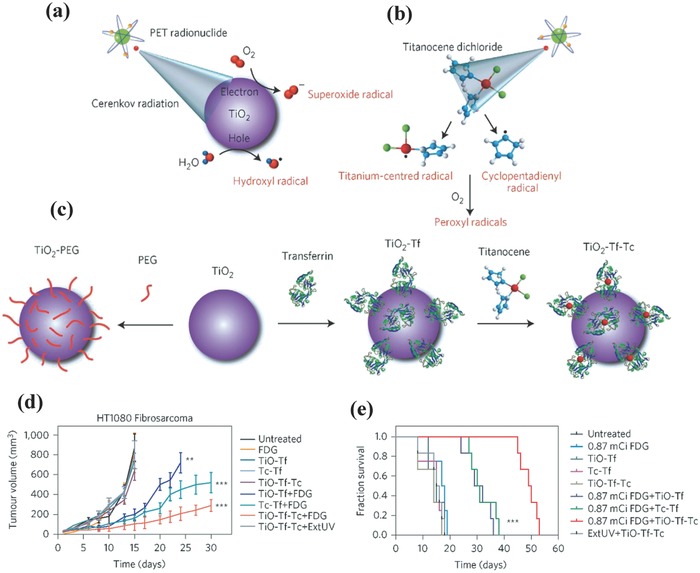
a) Schematic illustration of CR activation of TiO_2_ nanoparticles for producing cytotoxic hydroxyl and superoxide radicals by the electron–hole pair generation where CR was generated by PET radionuclides. b) The scheme of CR activation of Tc for the generation of cyclopentadienyl radical and titanium‐centered radical by photofragmentation. c) Schematic illustration of the fabrication of TiO_2_–PEG, TiO_2_–Tf, and TiO_2_–Tf–Tc, nanoparticles. d) The tumor‐volume changes of HT1080‐tumor‐bearing nude mice after varied treatments as indicated in the figure. e) The survival rate after the treatments with 0.87 mCi/0.1 mL FDG. Reproduced with permission.^174^ Copyright 2015, Springer Nature.

As an internal light source, CR is featured with high tissue‐penetrating depth, which is typically triggered when the charged particles (e.g., β^+^ and β^−^) pass through a dielectric medium beyond the light speed.^174^, ^175^, ^176^ The UV can be emitted by CR for triggering UV‐responsive photosensitizers for PDT.^174^, ^177^ On this ground, CR‐induced therapy was achieved by the radiation of PET radionuclides for activation of TiO_2_ nanoparticles to produce hydroxyl and superoxide radicals (**Figure** **10**a).^174^ Especially, titanocene (Tc) was further anchored onto the surface of TiO_2_ nanoparticles for enhancing and complementing CR‐irradiated TiO_2_ cytotoxicity because it could generate cyclopentadienyl and titanium‐centered radicals once exposure to UV light (Figure 10b). Furthermore, apo‐transferrin (Tf) was modified onto the surface of TiO_2_ nanoparticles for enhancing the positive accumulation into the tumor tissue (Figure 10c). The results demonstrated that the intravenous administration of Tf‐anchored TiO_2_ nanoparticles and clinically employed radionuclides efficiently suppressed the tumor growth (Figure 10d) accompanied with prolonged survival rate of tumor‐bearing mice (Figure 10e). This paradigm provides a new strategy to develop low‐radiance‐sensitive nanophotosensitizers for efficient Cerenkov‐radiation‐activated cancer therapy with the tissue‐depth impendence.

**Figure 11:**
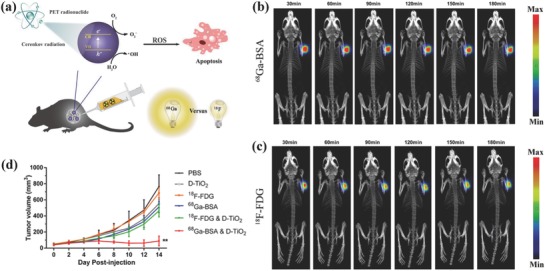
a) Schematic illustration of CR‐activated TiO_2_ photosensitizers for producing ROS to kill the cancer cells. The comparison of CR‐induced PDT by dynamic PET imaging of 4T1 tumor‐bearing mice after intratumor injection of equivalent amount of b) ^68^Ga‐BSA and c) ^18^F‐FDG from 30 min to 3 h. d) The tumor‐volume changes of tumor‐bearing mice after varied treatments as indicated in the figure. Reproduced with permission.^178^ Copyright 2018, American Chemical Society.

The efficient PDT strongly depends on the ROS production efficiency, which is significantly influenced by the local photon intensity.^179^ Gallium‐68 (Ga‐68) is a promising CR source because of the 30‐time higher Cerenkov productivity as compared to fluorine‐18 (F‐18) such as ^18^F‐fluorodeoxyglucose (^18^F‐FDG). Therefore, ^68^Ga‐labelled bovine serum albumin (^68^Ga‐BSA) was employed as the CR source to activate dextran‐modified TiO_2_ nanoparticles for inhibiting the tumor growth (**Figure** **11**a), which could emit UV light to produce electron (e^−^) and hole (h^+^) from energy band of TiO_2_ and generate ROS subsequently.^178^ By PET imaging, it has been found that intratumoral injection of ^68^Ga‐BSA and ^18^F‐FDG showed the similar tumor uptake of ^68^Ga‐BSA and ^18^F‐FDG (Figure 11b,c). Importantly, the tumor‐bearing mice after the treatment with ^68^Ga‐BSA and TiO_2_ photosensitizer exhibited significantly inhibited tumor volume (Figure 11d) and prolonged survival time while the mice in the group of ^18^F‐FDG and TiO_2_ showed much lower tumor‐suppressing rate, indicating that Ga‐68 could act as the more efficient radionuclide as compared to F‐18 for CR‐induced in vivo PDT on combating cancer. The effective cancer treatment of deep‐seated tumor by radiation‐activated TiO_2_ nanoparticles is highly promising for clinical use but the potential biosafety risk of radiation source should be seriously considered.

## 6 Ultrasound Irradiation on Titania for SDT

Ultrasound (US) has been broadly explored in biomedicine for decades, not only for diagnostic imaging but also for therapeutic applications.^180^, ^181^, ^182^, ^183^, ^184^, ^185^, ^186^ For instance, the thermal, mechanical, and cavitation effects of high‐intensity focused ultrasound (HIFU) have been used for noninvasive cancer surgery.^187^, ^188^, ^189^, ^190^ In addition, the sonosensitizer‐involved SDT produces ROS for inducing the cancer‐cell death for cancer‐dynamic therapy.^191^, ^192^, ^193^, ^194^, ^195^, ^196^, ^197^, ^198^ Especially, US is featured with high tissue‐penetrating depth in human bodies, which can reach internal organs such as liver, spleen, and kidney. Therefore, both the tissue‐penetrating capability and theranostic biosafety of US make it a promising exogenous physical triggering source for versatile biomedical applications.

As the mostly explored inorganic nanosonosensitizers with high biocompatibility and stability, titania nanoparticles have been extensively employed for US‐activated SDT against cancer.^199^, ^200^, ^201^, ^202^ PEGylated TiO_2_ nanoparticles have been demonstrated to be effective on inducing the cell death of U251 monolayer cells (1.0 MHz, 1.0 W cm^−2^), and the related therapeutic mechanism was found to be different from that of UV light‐induced PDT.^203^ Avidin protein‐conjugated TiO_2_ nanoparticles were designed to preferentially discriminate cancerous cells from healthy cells for targeted SDT.^204^ For in vivo assessment, the combination of TiO_2_ nanoparticles and US irradiation (1 MHz, 1.0 W cm^−2^, 2 min) substantially inhibited the tumor growth on subcutaneously implanted C32 xenograft, demonstrating the high in vivo therapeutic efficiency of TiO_2_‐sonosensitized SDT.^205^ Especially, the introduction of dual‐frequency US for activation of TiO_2_ nanoparticles as the nano‐sonosensitizers was demonstrated to be more efficient for enhancing the hydroxyl radical production, which was verified in vitro on killing HepG2 cells.^206^

**Figure 12:**
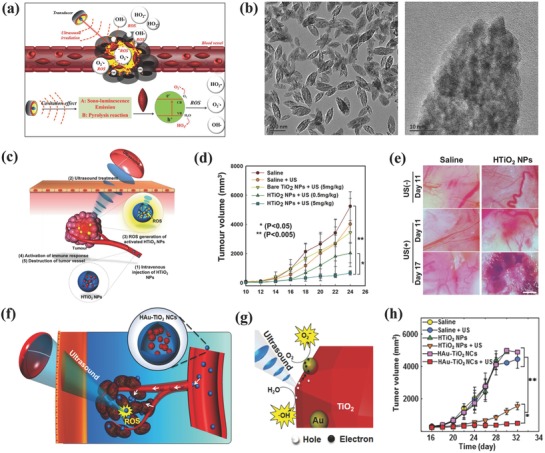
a) Schematic illustration of the accumulation of PEG–MTNs into the tumor tissue, and further US‐triggered production of ROS for killing cancer cells. b) TEM images of MTNs at low (left image) and high (right image) magnifications. Reproduced with permission.^84^ Copyright 2017, Royal Society of Chemistry. c) The scheme of HTiO_2_ nanoparticle‐enhanced SDT, including EPR effect‐enabled accumulation into tumor and US‐triggered ROS production to enhance the immune response and destroy tumor microvasculature. d) The tumor‐volume changes of SCC7 tumor‐bearing mice in each treatment group. e) The bright‐field images of tumor vasculature by US‐triggered SDT effect. Reproduced with permission.^207^ Copyright 2016, Springer Nature. f) Schematic illustration of in vivo US‐triggered activation of HAu–TiO_2_ nanoparticles for SDT and g) the underlying mechanism regarding the ROS production by US activation of HAu–TiO_2_ nanoparticles. h) The comparison of tumor‐volume changes with respect of feeding time by varied treatments as indicated in the figure. Reproduced with permission.^91^ Copyright 2016, American Chemical Society.

We recently synthesized MTNs for US‐triggered SDT (**Figure** **12**a).^84^ These MTNs were featured with ellipsoidal topology and high dispersity (Figure 12b). Especially, they showed the highly single‐crystalline structure with well‐defined mesoporosity, which could enhance the SDT efficiency based on the fact that the high crystallity without defects could avoid the recombination of electrons (e^−^) and holes (h^+^) as triggered by US irradiation. The mesoporosity potentially facilitated the encapsulation and delivery of therapeutic agents such as anticancer drugs. After accumulation into tumor tissue of PEGylated MTNs (PEG‐MTNs) via the typical enhanced permeability and retention (EPR) effect, the US‐triggered SDT effect achieved 40% tumor‐suppression rate under the intravenous administration mode.^84^ Hydrophilized TiO_2_ (HTiO_2_) nanoparticles were fabricated by anchoring CMD onto the surface of TiO_2_ nanoparticles for guaranteeing the high stability in physiological condition, prolonging the blood‐circulation duration and enhancing the tumor accumulation.^207^ The accumulation of HTiO_2_ into tumor tissue and further US activation not only enhanced the immune response but also destroyed the tumor microvasculature (Figure 12c), which was demonstrated by the gradually decreased tumor volume (Figure 12d) and the decreased tumor vasculature (Figure 12e) by US‐triggered SDT effect in bright‐field images.

It is noted that the low quantum yield of nanosonosensitizers resulting from the fast electron–hole recombination hinders the further clinical translation of TiO_2_‐based sonosensitizers. To address this critical issue, noble metal Au was combined with TiO_2_ nanoparticles to prevent the undesirable electron–hole recombination by trapping the sono‐excited electrons (Figure 12f,g).^91^ This principle has been extensively explored in the typical TiO_2_‐based photocatalysis. In addition, CMD was also anchored onto the surface of Au–TiO_2_ nanoparticles for further in vitro and in vivo evaluations. It is important to find that more ROS could be produced under US activation of Au–TiO_2_ composite nanosonosensitizers as compared to pure TiO_2_ without Au deposition, demonstrating the effectiveness of Au and TiO_2_ combination. This enhanced SDT effect was also revealed in tumor‐therapeutic outcome where the Au–TiO_2_ composite nanosonosensitizers induced the more significant tumor suppression as compared to TiO_2_ nanoparticles upon US activation (Figure 12h).^91^

**Figure 13:**
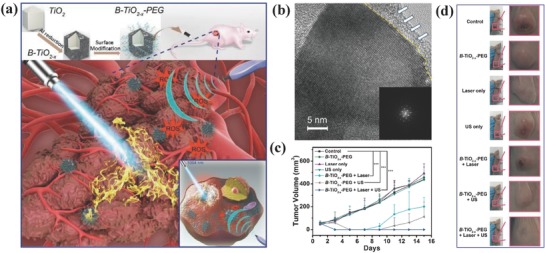
a) Schematic illustration of the fabrication of PEGylated B‐TiO_2−_ *_x_* nanosonosensitizers and enhanced SDT by ROS production and synergistic NIR‐II‐triggered photothermal hyperthermia. b) High‐resolution TEM image of B‐TiO_2−_ *_x_* nanosonosensitizers and corresponding SAED patter (inset image). c) The tumor‐volume changes of 4T1 tumor‐bearing mice after varied treatments as indicated in the figure, and d) corresponding photographic images of tumor at the end of treatments. Reproduced with permission.^85^ Copyright 2018, American Chemical Society.

By learning the lessons from typical photocatalysis, we recently fabricated an oxygen‐deficient TiO_2−‐_ *_x_* nanosonosensitizers for enhancing the SDT efficiency against tumor, which was achieved by Al reduction at high temperature to create an oxygen‐deficient TiO_2−_ *_x_* layer onto the surface of TiO_2_ nanoparticles (**Figure** **13**a,b).^85^ Such an oxygen‐deficient TiO_2−_ *_x_* layer facilitated and enhanced the separation of electrons (e^−^) and holes (h^+^) from the energy‐band of TiO_2_ semiconductor, which was activated by external physical US irradiation (Figure 13a). This effect has been demonstrated to substantially enhance the SDT efficiency at solvent level, in vitro cellular level and in vivo tumor xenograft level (Figure 13c). Especially, such a process to create oxygen‐deficient TiO_2−_ *_x_* (black TiO_2−_ *_x_*) endowed this unique TiO_2_‐based nano‐sonosensitizers with unique photothermal‐conversion capability at NIR‐II biowindow (1064 nm), which synergistically enhanced the SDT efficiency with the therapeutic outcome of complete tumor eradication (Figure 13c,d).^85^

Compared to light‐triggered TiO_2_‐based photosensitizer for PDT, US‐activated SDT based on TiO_2_ nano‐sonosensitizers is more applicable for clinical use based on the high tissue‐penetrating depth of US as compared to the conventional light as the irradiation source. However, US‐activated SDT is still at the preliminary stage, which still requires the further deep understanding of the underlying mechanism on the anticancer effect, which is highly beneficial for further improving the SDT efficiency on combating cancer.

## 7 Exogenous Physical Irradiation on Titania for Synergistic Cancer Therapy

Although above‐mentioned therapeutic modalities enabled by TiO_2_‐based nanoplatforms have shown promising clinical‐translation potential, each of these therapeutic modalities suffers from its intrinsic drawbacks hindering further broad applications. For instance, the therapeutic efficiency of RT, PDT, and SDT is limited by the hypoxia microenvironment of tumor. The heat shock response of local phototriggered hyperthermia causes the low PTT efficiency. The continuous chemotherapy usually induces the multidrug resistance (MDR) of cancer cells. To solve this critical issue, the combination therapy with involved two or more therapeutic modalities is expected to integrate the features and advantages of each therapeutic modality to achieve synergistic therapeutic outcome,^92^, ^208^, ^209^, ^210^, ^211^, ^212^, ^213^, ^214^ which has been broadly explored in abundant therapeutic‐modality combinations including physical‐triggering of TiO_2_ nanoplatforms.

**Figure 14:**
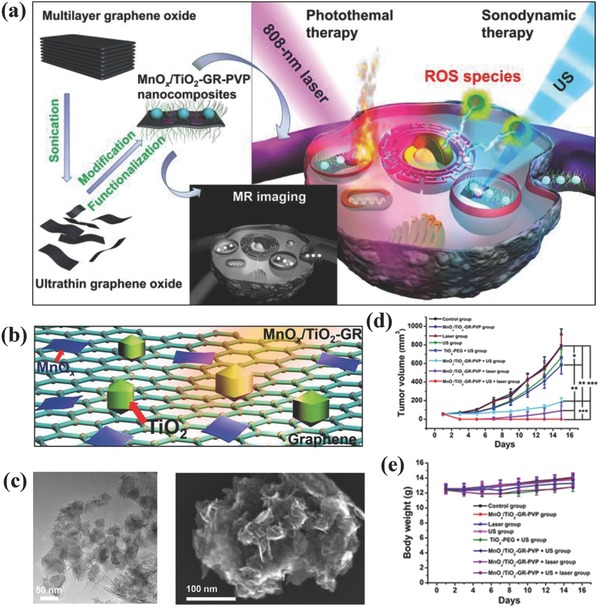
a) Schematic illustration of the fabrication of MnO*_x_*/TiO_2_–GR–PVP composite nanosheets, and their synergistic therapy based on MR/CT/PA multiple imaging‐guided photothermal hyperthermia (808 nm) and enhanced SDT. b) The scheme of loading MnO*_x_* and TiO_2_ onto the surface of graphene nanosheets, and corresponding c) TEM (left image) and SEM (right image) images. d) The tumor‐volume changes with the prolonged feeding time after varied treatments as shown in the figure, and e) corresponding body‐weight changes in each therapeutic group. Reproduced with permission.^90^ Copyright 2017, American Chemical Society.

Based on the high electroconductivity of graphene, we recently loaded TiO_2_ nanoparticles onto the surface of graphene (designated as MnO*_x_*/TiO_2_–GR–PVP, MnO*_x_* for MR imaging) for enhanced and synergistic SDT and photothermal hyperthermia (**Figure** **14**a), which was uniformly distributed onto graphene's surface (Figure 14b,c).^90^ On one hand, the high electroconductivity of graphene facilitates the separation of electron (e^−^) and hole (h^+^) pairs from the energy‐band structure of TiO_2_ nanosonosensitizers upon external US irradiation, which could significantly enhance the SDT efficiency on killing the cancer cells. On the other hand, the graphene matrix showed the high photothermal‐conversion performance for hyperthermia, which further synergistically enhanced the SDT efficiency of loaded TiO_2_ nanosonosensitizers (Figure 14d) with high therapeutic biosafety as indicated by neglectable body‐weight changes (Figure 14e). This paradigm demonstrates that the rational combination of nano‐TiO_2_ semiconductors with high electroconductivity nanosystems could enhance the SDT efficiency by facilitating the US‐triggered separation of electron and hole pairs.

**Figure 15:**
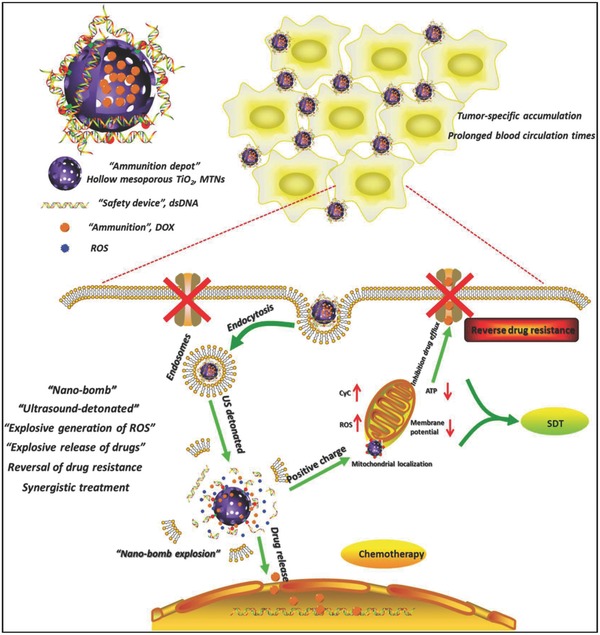
Schematic illustration of US‐triggered combinatorial therapy using a “nano‐bomb,” including US‐triggered SDT for ROS generation, US‐activated drug release, reversal of MDR, and final synergistic cancer treatment. Reproduced with permission.^215^ Copyright 2018, Elsevier.

To reverse the MDR of cancer cells, a “nano‐bomb” was designed for US‐triggered multiple and synergistic cancer therapy based on hollow MTNs.^215^ Chemotherapeutic drug doxorubicin acting as the ammunition was loaded into MTNs as the ammunition depot, and the surface of MTNs was coated by dsDNA as the safe device to avoid the prerelease of loaded doxorubicin (**Figure** **15**). Especially, the US irradiation on drug‐loaded MTNs achieved multiple effects, including US‐triggered SDT for MTN‐sonosensitized ROS generation, US‐activated drug release, reversal of MDR, and final synergistic cancer treatment. The reversal of MDR of MCF‐7/ADR cancer cells was based on the inhibition of mitochondrial energy supply by the US‐triggered “explosion” of MTNs, causing the substantially suppressed tumor growth.^215^

**Figure 16:**
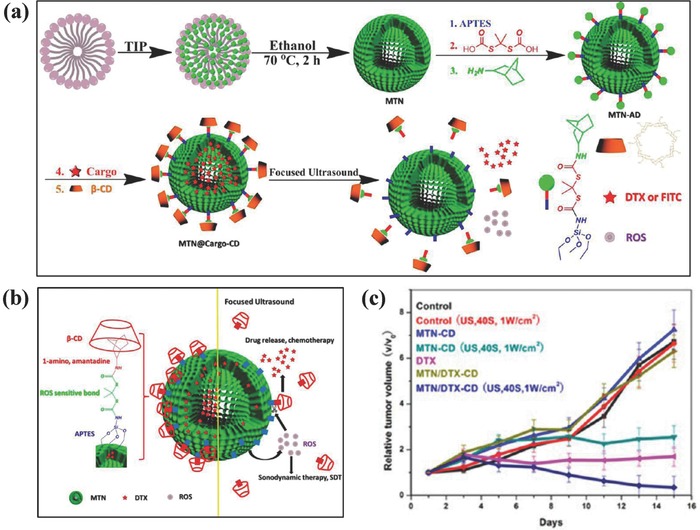
a) Schematic illustration of stepwise synthesis of MTN@DTX‐CD composite nanosystem. b) The scheme of microstructure of MTN@DTX‐CD and the functionality of each component. c) The tumor‐volume changes of S180 tumor‐bearing mice after varied treatments as indicated in the figure. Reproduced with permission.^216^ Copyright 2015, American Chemical Society.

In addition, envelope‐type mesoporous titanium dioxide nanoparticles (MTN) were fabricated with the subsequent loading of docetaxel (DTX) accompanied with a high drug‐loading capacity of ≈26% (**Figure** **16**a).^216^ Furthermore, β‐cyclodextrin (β‐CD) was anchored onto the surface of MTN as a bulky gatekeeper, which was based on a ROS‐sensitive linker to seal DTX within the mesopores (Figure 16b). Upon US irradiation, large amounts of ROS were produced by SDT effect to break the ROS‐sensitive linker and then trigger the DTX release from the mesopores. Therefore, the US irradiation not only induced ROS generation for SDT cancer therapy, but also triggered DTX releasing from mesopores for synergistic chemotherapy, which was demonstrated by the synergistic therapeutic outcome where the tumor growth in the synergistic group got the maximum suppression (Figure 16c).^216^ Magnetic core/shell structured Fe_3_O_4_‐NaYF@TiO_2_ nanocomposites were constructed for synergistic chemotherapy by loaded doxorubicin and SDT by the TiO_2_ component.^217^ The further surface engineering with hyaluronic acid (HA) enabled targeted intracellular transportation, which induced high tumor‐inhibition rate of 88.36% in synergistic group, much higher than that of the single therapeutic modality such as chemotherapy (28.36%) and SDT (38.91%).

TiO_2_‐based nanoparticles have been explored as the drug‐delivery nanosystems for chemotherapy.^218^, ^219^, ^220^, ^221^ For instance, ZnPc@TiO_2_ (ZnPc: zinc phthalocyanine) hybrid nanoparticles were employed for the intracellular delivery of anticancer drug doxorubicin based on the electrostatic interaction between drug molecules and nanocarriers.^67^ Doxorubicin was also loaded into Fe_3_O_4_@TiO_2_ core/shell nanoparticles for synergistic chemotherapy by loaded chemotherapeutic drug and US‐activated SDT by the TiO_2_ component as sonosensitizer.^222^ The typical strategy only loaded the chemotherapeutic drug molecules onto the surface of TiO_2_ nanoparticles, therefore the drug‐loading amount was low and the release in uncontrollable. On this ground, Wu and co‐workers coated a mesoporous silica layer onto the surface of black TiO_2_ nanoparticles for achieving synergistic chemotherapy and photothermal hyperthermia.^223^ On one hand, the mesoporous silica layer provided the large reservoir for the drug loading/delivery. On the other hand, the black TiO_2_ acted as the photothermal‐conversion nanoagents for PTT. By the integration with folic‐acid targeting, the mesoporous silica‐coated black TiO_2_‐enabled produced the synergistic therapeutic outcome on suppressing the tumor growth against MCF‐7 breast cancer xenograft.^223^

**Figure 17:**
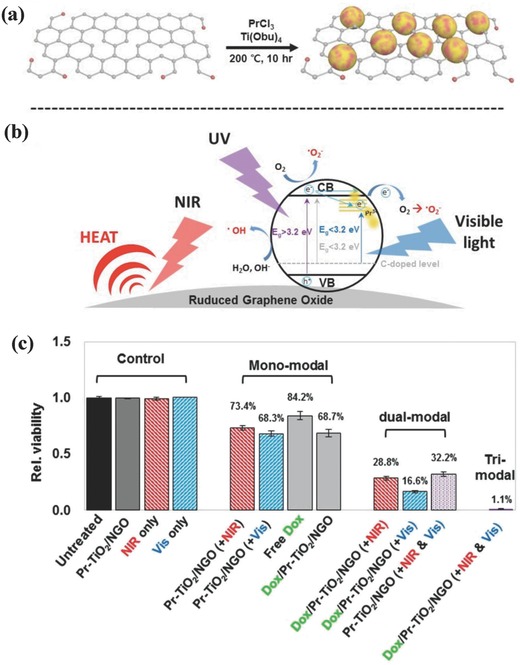
The scheme of a) synthesizing Pr‐TiO_2_/NGO nanocomposites and b) light‐activated photocatalytic process for ROS production. c) In vitro therapeutic efficacy of different treatments, including mono, dual, and triple‐modality cancer‐cell treatment such as chemotherapy (loaded Dox), PTT, and phototriggered dynamic therapy. Reproduced with permission.^224^ Copyright 2015, WILEY‐VCH Verlag GmbH & Co. KGaA, Weinheim.

TiO_2_ nanoparticles could integrate with other functional nanosystems for achieving some specific synergistic therapeutic purposes. For instance, praseodymium (Pr)‐doped TiO_2_ on GO nanoplatforms were fabricated by a facile hydrothermal synthesis (**Figure** **17**a).^224^ First, the sp^2^ carbonaceous framework of GO converted NIR light into heat for photothermal hyperthermia (Figure 17b). Second, the Pr‐doped TiO_2_ nanoparticles could absorb more hydroxide ions onto the surface to promote the generation of hydroxyl radicals and suppress the electron–hole recombination. Third, the 4f electron transition of doped Pr achieved the incorporation of additional energy levels in the bandgap of TiO_2_, which induced the enhanced photocatalytic activity on killing cancer cells under visible light (450 nm). Fourth, this composite nanosystem could store therapeutic anticancer agents (doxorubicin) for enhanced chemotherapy. Especially, the synergistic chemotherapy, PTT and phototriggered PDT (triple‐therapeutic modality) significantly induced the cancer‐cell death as compared to either monomodal or dual‐modal therapy (Figure 17c).^224^

**Figure 18:**
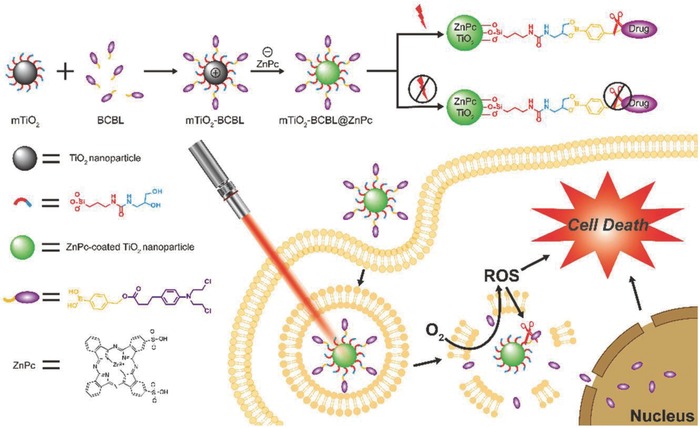
Schematic illustration of synthesizing mTiO_2_–BCBL@ZnPc nanoparticles and their unique therapeutic functionality for cancer therapy, which was based on ROS‐responsive drug‐releasing performance under NIR irradiation by breaking up the phenylboronic acid ether. Reproduced with permission.^225^ Copyright 2018, Royal Society of Chemistry.

The previous discussion has mentioned that the UV wavelength range of 320 to 400 nm might cause the phototoxicity and have the low‐penetrating capability. To solve the critical issue of UV‐responsiveness of traditional TiO_2_ nanoparticles, zinc phthalocyanine as the intriguing photochemical molecule with high stability, efficiently extended the light window of TiO_2_ nanoparticles from UV region to NIR region for phototreatment, which was based on an intercomponent electron transfer between zinc phthalocyanine and titania nanoparticles.^225^ Especially, the ROS‐sensitive compound BCBL was conjugated to zinc phthalocyanine‐modified TiO_2_ nanoparticles for ROS‐triggered chemotherapy (**Figure** **18**). Upon NIR irradiation, the generated large amounts of ROS triggered the release of loaded BCBL for chemotherapy, which also acted as the toxic species for PDT, inducing the synergistic chemotherapeutic and PDT efficiency.^225^ The UV‐activated TiO_2_ nanoparticles have been previously demonstrated to reverse the MDR of cancer cells.^226^ To further overcome critical issue of UV light for reversal of MDR, doxorubicin was loaded into NaYF_4_:Yb/Tm‐TiO_2_ inorganic photosensitizers for simultaneous 980 nm NIR‐activated PDT and intracellular drug delivery.^227^ The surface folic acid modification enhanced intracellular uptake of the nano‐photosensitizer and accelerated the doxorubicin release in both drug‐sensitive MCF‐7 and drug‐resistant MCF‐7/ADR cancer cells, inducing the synergistic MCF‐7/ADR tumor‐inhibition rate of up to 90.33%, significantly higher than that of free doxorubicin.

**Figure 19:**
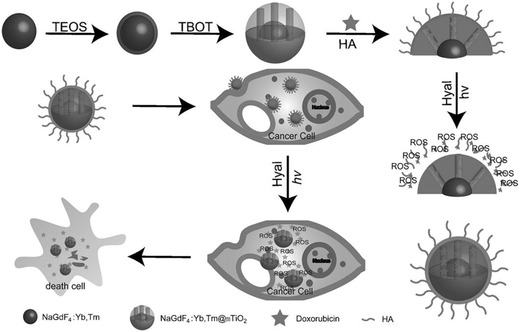
The scheme of the fabrication of NaGdF_4_:Yb,Tm@mTiO_2_ core/shell nanoparticles the novel drug‐delivery system for simultaneous and synergistic chemotherapy and NIR‐triggered PDT. Reproduced with permission.^228^ Copyright 2014, WILEY‐VCH Verlag GmbH & Co. KGaA, Weinheim.

The construction of mesoporous titania‐coated UCNPs is expected to achieve NIR‐triggered PDT and simultaneous chemotherapy for synergistic therapy, originating from UNCPs core and mesoporous titania shell. Mesoporous TiO_2_ upconverting nanoparticles (abbreviated as MTUN) were synthesized by direct coating of a mesoporous TiO_2_ layer onto the surface of NaGdF_4_:Yb25%,Tm0.3% as mediated by a middle silica layer (**Figure** **19**).^228^ The UCNPs converted NIR irradiation to UV light, which further activated mesoporous TiO_2_ layer to generate ROS for inducing cancer‐cell apoptosis. Especially, the well‐defined mesopores of surface TiO_2_ layer acted as the drug‐storage reservoirs for drug delivery and chemotherapy, inducing the synergistic NIR‐activated PDT and chemotherapy. Importantly, the HA was anchored onto the surface of this composite nanosystem for targeting cluster determinant 44 (CD44) that was overexpressed on cancer‐cell membrane and achieving controlled drug releasing as triggered by the specific enzyme in tumor region. Based on mesoporous TiO_2_‐coated UCNPs, a photolabile *o*‐nitrobenzyl derivative was incorporated to act as the gate by forming a sensitive linker for avoiding the drug releasing.^229^ The NIR‐triggered ROS production not only induced the PDT effect, but also cause the breaking of the sensitive linker for on‐demand drug releasing, leading to synergistic chemotherapy and PDT against cancer cells. To further enhance the drug‐loading capability, rattle‐type UCNPs@Void@TiO_2_ nanocomposites were fabricated with large voids between UCNPs core and mesoporous TiO_2_ shell, producing TiO_2_‐based PDT by NIR irradiation and doxorubicin‐induced synergistic chemotherapy.^230^

**Figure 20:**
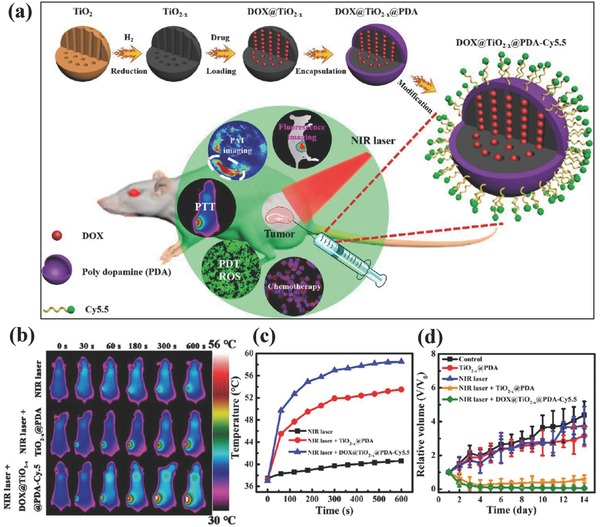
a) Schematic illustration of synthesizing DOX@TiO_2−_ *_x_*@PAD‐Cy5.5 nanocomposites, and their unique functionality for dual‐mode imaging‐guided synergistic chemotherapy, PTT, and PDT. b) Infrared thermal images of MDA‐MB‐231 tumor‐bearing mice after the administration of DOX@TiO_2−_ *_x_*@PAD‐Cy5.5 nanocomposites followed by NIR irradiation, and c) corresponding temperature‐elevating profiles. d) The tumor‐volume change as a function of feeding time after the different treatments as indicated in the figure. Reproduced with permission.^86^ Copyright 2017, American Chemical Society.

The rational structure design of TiO_2_‐based nanoplatforms could endow them with more therapeutic functionalities.^231^ It has been demonstrated that anticancer drug doxorubicin‐loaded TiO_2_ nanoparticles overcame the MDR of breast cancer cells (MCF‐7/ADR) by bypassing the P‐glycoprotein‐mediated doxorubicin‐pumping system.^218^ Furthermore, TiO_2_‐based composite nanosystems (DOX@TiO_2−_ *_x_*@PAD‐Cy5.5, PDA: poly dopamine) were stepwise synthesized for simultaneous fluorescent/PAT bimodal tumor imaging and NIR‐activated chemo/photodynamic/photothermal combinatorial therapy (**Figure** **20**a).^86^ Because of the high photothermal‐conversion capability of TiO_2−_ *_x_* matrix, the tumor temperature was rapidly elevated upon NIR irradiation (808 nm, Figure 20b,c). Especially, the NIR irradiation of DOX@TiO_2−_ *_x_*@PAD‐Cy5.5 generated ROS for efficient PDT, and the presence of mesopores in TiO_2−_ *_x_* matrix provided the reservoirs for the encapsulation and controllable delivery of therapeutic anticancer drugs (doxorubicin) with unique responsiveness to endogenous mild acidity of TME and exogenous NIR irradiation. The simultaneous and synergistic triple therapy induced the high tumor‐suppressing outcome with almost complete tumor eradication (Figure 20d), which was caused by DOX‐induced DNA damage and PDT/PTT‐induced mitochondrial dysfunction/change of membrane.^86^

Both the microstructure and functionality of TiO_2_‐based nanoplatforms could be rationally designed for achieving synergistic cancer‐therapeutic outcome, which is highly unique in TiO_2_ nanosystems because of their easy synthesis and specific semiconductor nature. It should be noted that the surface inertness of TiO_2_ nanoparticles makes their surface engineering difficult, which typically seeks the help from other organic or inorganic functionality by some unique synthetic strategies, such as inorganic silica coating or hydrophobic–hydrophobic interaction. It should be noted that the multifunctionalization design of TiO_2_ nanoparticles should be based on the practical requirements of clinical use because the complex design of these composite nanosystems usually causes the difficulty for potential clinical translation.

## 8 Diagnostic‐Imaging of Titania for Therapeutic Guidance and Monitoring

It has been well demonstrated that some metal oxides nanoparticles can act as the contrast agents for enhancing the diagnostic‐imaging resolution and sensitivity of diverse imaging modalities, such as manganese oxide (T_1_‐weighted MR imaging),^233^, ^234^, ^235^, ^236^, ^237^, ^238^ gadolinium oxide (T_1_‐weighted MR imaging),^239^, ^240^, ^241^, ^242^ iron oxide (T_2_‐weighted MR imaging),^243^, ^244^, ^245^, ^246^, ^247^, ^248^ and tantalum oxide (CT imaging).^249^, ^250^, ^251^ Titania nanoparticles have been seldom explored for enhancing the contrast of various diagnostic‐imaging modalities because of lacking the characteristic physiochemical properties. Fortunately, the fast advances of material‐synthetic chemistry and nanomedicine make it possible based on two typical strategies. On one hand, the structure of titania nanoparticles can be tuned with contrast‐enhanced imaging functionality. On the other hand, these titania nanoparticles can be integrated with some imaging contrast agents for achieving some specific imaging purposes. It is intriguing that the diagnostic‐imaging capability of titania nanoparticles can play the specific role for precise therapeutic guidance and monitoring, which is promising for enhancing the therapeutic efficiency and mitigating the damage to the surrounding normal tissue/cell.

**Figure 21:**
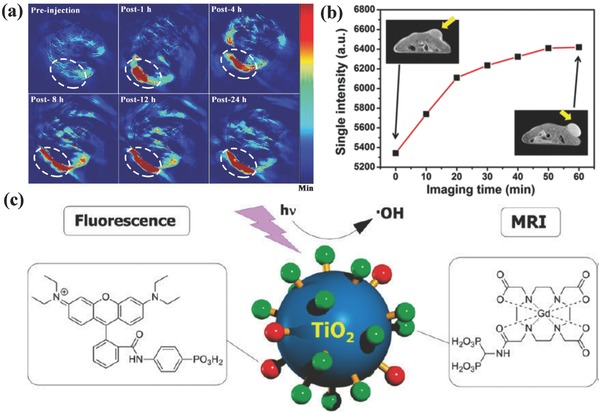
a) In vivo PA imaging of tumor‐bearing mice before and after intratumoral administration of DOX@TiO_2−_ *_x_*@PDA‐Cy5.5 nanocomposites for prolonged durations. Reproduced with permission.^86^ Copyright 2017, American Chemical Society. b) In vivo T_1_‐weighted MRI signal‐intensity variations after intravenous administration of MnO*_x_*/TiO_2_–GR–PVP for different time intervals. Reproduced with permission.^90^ Copyright 2017, American Chemical Society. c) Schematic illustration of surface conjugation of fluorescent moieties and Gd chelates for concurrent fluorescence and MR imaging, and the further UV‐activated production of hydroxyl radicals for killing the cancer cells. Reproduced with permission.^232^ Copyright 2011, American Chemical Society.

The aforementioned discussion has revealed that the oxygen‐deficient black titania nanoparticles could be endowed with photothermal‐conversion capability at NIR range. This property has been generally developed for contrast‐enhanced PA imaging, such as 2D MXene,^130^ black phosphorous,^252^, ^253^ MoS_2,_ 254, 255 and Au nanoparticles.^256^, ^257^, ^258^ On this ground, oxygen‐deficient black titania nanoparticles were explored as the contrast agents for PA imaging after the injection into tumor‐bearing mice.^86^ It has been found that the obvious contrast enhancement was observed in tumor region after intratumoral injection of these black titania nanoparticles (**Figure** **21**a), demonstrating their imaging capability. As another paradigm, titania nanoparticles were integrated with manganese oxide nanoparticles to construct a composite nanosystem, where the integrated manganese oxide nanoparticles acted as the contrast agents for T_1_‐weighted MR imaging and guided the SDT of cancer as contributed by the titania component in the composite nanosystem (Figure 21b).^90^ Especially, titania nanoparticles were simultaneously conjugated with fluorescent moieties and Gd‐based chelates for labeling HeLa cancer cells by both fluorescence microscopy and MR imaging, showing the dual‐imaging capability of the titania‐based composite nanosystem (Figure 21c).^232^ Additionally, the construction of magnetic Fe_3_O_4_–TiO_2_ nanocomposites achieved simultaneous T_2_‐weighted MR imaging and PDT against MCF‐7 cancer cells.^259^

## 9 Biocompatibility and Biosafety of Theranostic Titania

The previous discussion has mentioned that titanium (Ti) element is one of the most biocompatible elements present in nature, as demonstrated by the fact that TiO_2_‐based micro/nanoparticles have been broadly used in food, cosmetics, and sunscreen and Ti‐containing metal alloys has been used as the medical implantation devices. It is highly expected that these Ti‐based nanoparticles as discussed in the review are also biocompatible in biomedical applications. However, it is generally accepted that the particles would induce some abnormal biological behaviors and effects or even toxicity when their particle size are reduced into nanoscale. Therefore, systematic investigation of these titania nanoparticles should be further conducted to guarantee their high biocompatibility and biosafety for further clinical translation.

Actually, the biological effects and biocompatibility of titania‐based compound or micro/nanoparticles have been broadly investigated in the past decade,^260^, ^261^, ^262^, ^263^, ^264^, ^265^, ^266^, ^267^, ^268^, ^269^, ^270^, ^271^, ^272^, ^273^, ^274^, ^275^, ^276^ which has also been summarized and discussed in some excellent reviews.^277^, ^278^, ^279^ Therefore, this review herein focuses more on the biocompatibility and biosafety of some rationally designed novel titania‐based nanoplatforms with unique responsiveness to exogenous physical irradiations. Our previous work has demonstrated the SDT effect of MTNs with well‐defined mesopores for combating cancer.^84^ Furthermore, we systematically assessed the in vivo biocompatibility of these MTNs on healthy mice. It has been found that either single high dose at 150 mg kg^−1^ or repeated dose at as high as total 400 mg kg^−1^ exhibited no obvious in vivo toxicity, as demonstrated by the hematology markers and blood biomedical parameters where no significant changes were monitored as compared to control groups without any treatments, indicating the high biocompatibility of these MTNs.^84^ For titania‐based nanocomposites, it has been demonstrated that Au_25_/B‐B‐TiO_2−_ *_x_* nanotubes not only showed low hemolytic effect on red blood cells, but also revealed their low cytotoxicity to L929 cells (mouse fibroblast cell line) and HeLa cells (human cervical cancer cell line).^119^ The targeting titania‐based nanocomposites anti‐EGFR–PEG–TiO_2_–UCNPs were demonstrated to have no major sub‐acute or long‐term toxicity as revealed in no significant blood biomedical, hematological or histopathological changes at the dose of 50 mg kg^−1^.^104^

One of the unique advantages of titania‐based nanoplatforms with responsiveness to exogenous physical irradiation is the high therapeutic biosafety. These nanoplatforms can only induce the toxic effect under the tumor sites as irradiated by external diverse physical triggers while other organs or tissues without physical irradiation will not be damaged even if these titania nanoparticles are accumulated into them. This high therapeutic biosafety is expected to significantly mitigate the side effects of traditional therapeutic modalities such as chemotherapy where the toxic drugs or substances are usually introduced, causing the severe side effects. Our results have demonstrated that SDT against cancer with the assistance of black TiO_2−_ *_x_* nanosonosensitizers induced no obvious pathological changes of the major organs after the therapeutic process, demonstrating the high therapeutic biosafety of this SDT modality.^85^ In addition, the combinatorial and synergistic SDT and chemotherapy of DTX‐loaded MTN were demonstrated to be featured with sustainably decreased side effects of loaded chemotherapeutic drug DTX by avoiding the spleen and hematologic toxicity to tumor‐bearing mice.^216^

## 10 Conclusions and Outlook

As one of the mostly explored biocompatible metal oxides in biomedicine, TiO_2_ nanosystems are featured with their intrinsic physiochemical properties for some specific theranostic applications, which is mainly originated from their semiconductor nature. Traditional strategies mainly focus on the UV light irradiation of TiO_2_ nanoparticles for PDT by forming the electrons (e^−^) and holes (h^+^) pairs from the energy‐band structure and then inducing the ROS generation for killing the cancer cells. The potential phototoxicity and low tissue‐penetrating depth of UV light severely limit the further in vivo biomedical applications of these TiO_2_ nanosystems. The fast development of nanosynthetic material chemistry enables the fine tuning of the composition, nanostructure, and property of TiO_2_ nanosystems possible. Importantly, the intriguing development of theranostic nanomedicine promotes the generation of diverse novel therapeutic modalities, which can be easily extended to TiO_2_ nanosystems for achieving physiochemical property‐oriented bioapplications, especially for cancer treatment. On this ground, this review mainly focuses on the very‐recent development of TiO_2_‐based nanoplatforms for cancer treatment with specific focuses on the NIR‐triggered photothermal hyperthermia, NIR‐activated PDT, X‐ray/CR‐activated deep‐seated PDT, US‐triggered sonodynamic therapy, and some synergistic therapeutic paradigms. Most of these novel therapeutic modalities are based on the semiconductor nature of TiO_2_ nanoplatforms, together with the defect modulation for PTT (**Table** **1**).

**Table 1:** Paradigms of nanotitania semiconductors for exogenous physical irradiation‐activated tumor‐specific therapy

Nanotitania	Irradiation source	Therapeutic modality	Performance	Refs.
Targeted TiO_2_	Visible light	Photodynamic therapy	Enhanced intracellular uptake and visible light‐activated PDT for damaging the cell membrane	109
N3‐TiO_2_	Light irradiation (365 nm)	Photodynamic therapy	Enhanced hydroxyl radical production under the hypoxic condition	111
UCNPs@TiO_2_	NIR irradiation (980 nm)	Photodynamic therapy	Inducing the substantial tumor suppression with high therapeutic efficiency by NIR irradiation	88
UCNPs@TiO_2_	NIR irradiation (980 nm)	Photodynamic therapy	Efficiently killing the cancer cells both in vitro and in vivo by NIR activation	87
Au_25_/B–TiO_2−_ *_x_*	NIR irradiation (650 nm)	Photodynamic therapy	Improved tumor‐suppressing effect based on photocatalytic synergistic effect by NIR irradiation	119
TiO_2_–Au–graphene	Simulated sunlight	Photodynamic therapy	Triggering a series of toxicological effects on killing B16F1 melanoma cells against B16F1 tumor xenograft	121
Black TiO_2−_ *_x_*	NIR irradiation (808 nm)	Photothermal therapy	Elevating the tumor temperature and inducing tumor‐tissue hyperthermia	147
Nb‐doped TiO_2_	NIR irradiation (1064 nm)	Photothermal therapy	Ablating the tumor tissue and suppressing tumor growth at NIR‐II biowindow	148
Au–TiO_2_	X‐ray	Photodynamic therapy	Inducing a synergistic therapeutic outcome with high tumor‐suppressing effect and improved survival rate of mice	92
TiO_2_–Tc‐Tf	Cerenkov radiation	Photodynamic therapy	Suppressing tumor growth and improved survival rate with deep tissue‐penetrating depth	174
Dextran–TiO_2_	Cerenkov radiation	Photodynamic therapy	Efficiently killing the cancer cells and improving the survival rate of tumor‐bearing mice	178
Mesoporous TiO_2_	Ultrasound	Sonodynamic therapy	Inducing tumor‐suppressing effect against 4T1 tumor xenograft	84
Hydrophilized TiO_2_	Ultrasound	Sonodynamic therapy	Enhancing immune response, suppressing tumor growth and destroying tumor microvasculature	207
Au–TiO_2_	Ultrasound	Sonodynamic therapy	Improved SDT effect against cancer by trapping the sono‐excited electrons	91
Black TiO_2−_ *_x_*	Ultrasound and NIR (1064 nm)	Sonodynamic therapy and photothermal therapy	Synergistic SDT and PTT on killing the cancer cells accompanied by enhanced SDT effect by oxygen‐deficient titania layer	85
MnO*_x_*/TiO_2_‐GR	Ultrasound and NIR (808 nm)	Sonodynamic therapy and photothermal therapy	Decreasing the re‐combination of electrons and holes for enhanced SDT effect on suppressing the tumor growth	90
Targeted TiO_2_	Visible light	Photodynamic therapy	Enhanced intracellular uptake and visible light‐activated PDT for damaging the cell membrane	109

**Figure 22:**
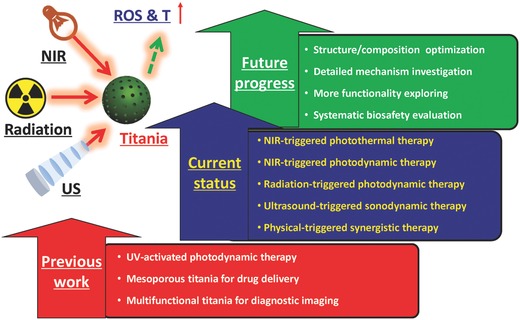
The summative scheme of previous work, current status, and future progress on physical irradiation‐activated titania nanoparticles for versatile biomedical applications, especially on combating cancer.

The unique physiochemical property of TiO_2_ nanosystems has achieved high therapeutic efficacy of aforementioned cancer‐therapeutic modalities, which is difficult to be achieved in other metal oxides such as SiO_2_ nanoparticles and superparamagnetic Fe_3_O_4_ nanosystems. Although these TiO_2_‐based novel therapeutic modalities are highly promising, it should be noted that they are still in infancy and at the preliminary stage. The further clinical translation is still facing some critical challenges to be resolved in near future as discussed in detail in the following subsections (**Figure** **22**).

### 10.1 Fabrication of TiO_2_ Nanosystems

The synthetic process for desirable TiO_2_ nanosystems is a bit more difficult as compared to other metal oxides such as SiO_2_ and Fe_3_O_4_ because the hydrolysis of titanium precursors is very fast in most cases. Therefore, their morphology and nanostructure are difficult to be precisely controlled. Especially, some fabrication process requires high‐temperature treatment such as the metal reduction to synthesize oxygen‐deficient black TiO_2−_ *_x_* nanoparticles, which avoidably causes the aggregation of TiO_2_ nanoparticles with low dispersity. In addition, there lacks the specific surface chemistry for the surface modification of these TiO_2_ nanoparticles, which, however, is necessary for guaranteeing their high stability in physiological solution or achieving the high tumor accumulation by targeting modification. These fabrication difficulties might lower the therapeutic efficacy of TiO_2_‐based therapeutic modalities or cause the biosafety issue. Therefore, two strategies are suggested to fabricate desirable TiO_2_ nanosystems. On one hand, the precise controlling of the sol–gel process during the synthesis of TiO_2_ nanosystems should be taken into consideration, which can fabricate TiO_2_ nanoparticles with desirable composition, nanostructure, and physiochemical property. On the other hand, the surface of as‐synthesized TiO_2_ nanoparticles could be endowed with some functional groups for further surface modification, which can be achieved by some specific treatments such as PEGylation or silica/mesoporous silica coating. It means that the surface chemistry can be controlled by using some “mediators” for satisfying the surface‐modification requirements.

### 10.2 Biocompatibility Issue of TiO_2_ Nanosystems

As compared to SiO_2_ and Fe_3_O_4_ metal oxides, the biological effects and biocompatibility of TiO_2_ nanoparticles are significantly less explored, which severely hinders their further clinical translation because of the lack of solid biocompatibility data. It is considered that TiO_2_ has been used in colorant in food, cosmetics and sunscreen and Ti‐containing metal alloys has been broadly used as the medical implantation devices, therefore these TiO_2_ nanosystems might possess the relatively high biocompatibility. Our previous results have demonstrated the low in vivo toxicity of either mesoporous TiO_2_ nanoparticles or black TiO_2−_ *_x_* nanosystems. These preliminary results are encouraging, but the further systematic investigations on the biocompatibility and biosafety issue are still highly urgent and necessary, which is the following research target in the near future.

### 10.3 Not Very Clear Mechanism on TiO_2_‐Based Novel Therapeutic Modalities

To overcome the drawbacks of traditional UV light irradiation for activating TiO_2_ nanoparticles, NIR light, X‐ray, CR, and US have recently been explored to activate TiO_2_ nanoparticles. The therapeutic performance is highly encouraging, but the underlying mechanism has not been fully revealed. Most of the results are mainly based on some phenomena in vitro. The exact in vivo therapeutic process is still highly challenging to monitor and determine because of the lack of adequate techniques and the complex in vivo environment, making it difficult to further optimize and enhance the therapeutic efficacy due to the lack of precise knowledge on the related mechanism. Therefore, the further therapeutic‐efficacy optimization requires the knowledge accumulation of the therapeutic mechanism, which is highly difficult but significantly urgent.

### 10.4 Influence of Crystalline Types of TiO_2_ Nanoparticles on Therapeutic Performance

It has been well documented that TiO_2_ nanoparticles have varied crystalline types with different physiochemical properties. However, at current stage, most therapeutic applications of titania nanoparticles did not consider the influences on the crystalline types of titania nanoparticles because the therapeutic use of titania nanoparticles as the emerging inorganic nanoplatform is still in the infancy, which still requires the following systematic investigations on the detailed underlying mechanism of the influence of crystalline types, precise structure/composition control and the following performance optimization.

### 10.5 More Biomedical Applications and Close Collaborations

Most of the therapeutic applications of TiO_2_‐based nanoparticles are related to cancer treatment except the antibacterial applications in some specific conditions. The intriguing performances of TiO_2_ nanoparticles with unique responsiveness to some types of external irradiations provide more opportunities for broader biomedical applications, such as tissue engineering, wound healing, gene therapy, stem‐cell therapy, etc. Therefore, the following fundamental researches should focus more on other specific bioapplications. However, it should be noted that such a multidisciplinary research requires the close collaborations of researchers/scientists with different background, which can guarantee each step of the clinical translation of TiO_2_‐based nanosystems is involved with professional researchers/experts with adequate knowledge to deal with the critical issues during the translation.

TiO_2_‐based nanoplatforms have emerged as one of the most promising therapeutic nanplatforms for cancer treatment because of their intrinsic physiochemical property and relatively high biocompatibility, which might be potential to act as the alternative substitute for mostly explored SiO_2_ and Fe_3_O_4_ metal oxides. This intriguing expectation should come true after further systematic investigations on their fabrication, biosafety evaluation and performance optimization. Especially, the nanomedical applications of TiO_2_‐based nanoplatforms also inspire the researchers to explore more functional inorganic nanosystems with some unique property‐oriented biomedical applications. Therefore, it is highly believed that these TiO_2_‐based nanoplatforms will find their broad bioapplications in theranostic nanomedicine and personalized biomedicine based on the fast developments of material science, chemistry and the multidisciplinary theranostic nanomedicine.

## Conflict of Interest

The authors declare no conflict of interest.
